# Supplementary material for: Aedes mosquitoes acquire and transmit Zika virus by breeding in contaminated aquatic environments
Source: Nat Commun. 2019 Mar 22;10:1324. doi: 10.1038/s41467-019-09256-0 (PMC6430813; doi:10.1038/s41467-019-09256-0)
Supplement: Supplementary file 1 — Supplementary Information [file 41467_2019_9256_MOESM1_ESM.pdf]

Supplementary Information

Aedes mosquitoes acquire and transmit Zika virus by breeding in contaminated aquatic environments

Du et al., 2019

Supplementary Information

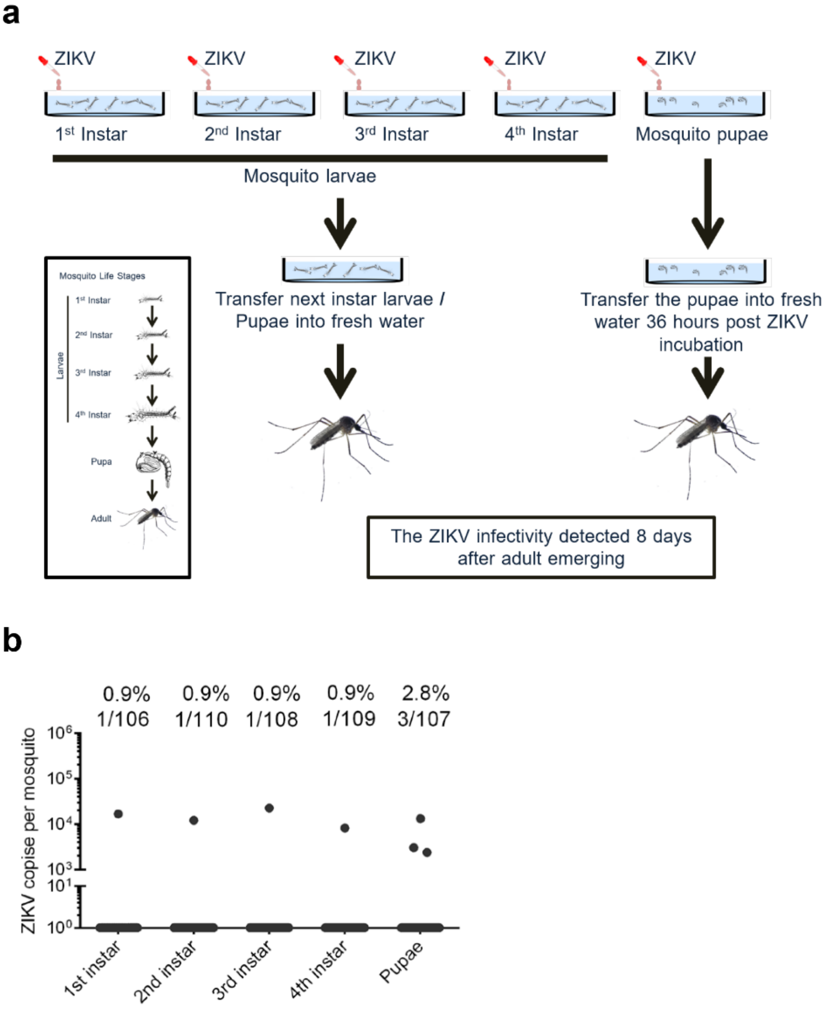

Supplementary Fig. 1 Assessing ZIKV infection in mosquitoes breeding from the diluted ZIKV urine

a Schematic representation. The ZIKV urine (from Donor 3, final titer 20 pfu/ml) was used for *A. aegypti* Rockefeller strain larvae and pupae breeding. After development to the next stage, the larvae or pupae were then transferred to a new container with fresh water. The emerging adult mosquitoes were reared for an additional 8 days for ZIKV detection by RT-qPCR.

b ZIKV infection in mosquitoes breeding from diluted ZIKV urine. The emerging adults were reared for 8 days for ZIKV detection by RT-qPCR. The number of infected mosquitoes relative to the total number of mosquitoes is shown at the top of each column. One dot represents a mosquito. The percentages are represented as the ratios of mosquito infection. The data were pooled from 3 independent biological replicates.

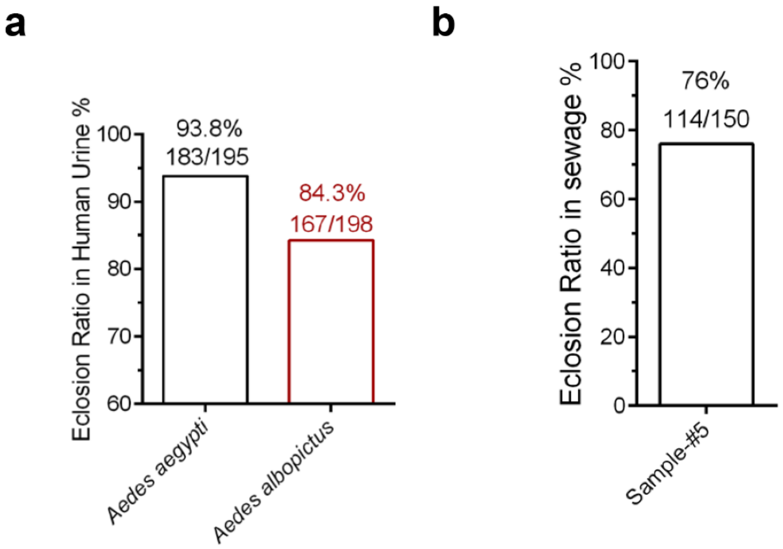

Supplementary Fig. 2 Eclosion ratio of mosquito pupae in human urine and sewage sample.

The freshly emerging pupae of various mosquito species were reared in human urine (*A. aegypti* Rockefeller strain and *A. albopictus* Jiangsu strain) (**a**) or sewage sample (*A. aegypti* Brazil Paraiba strain) (**b**). The eclosion ratio of mosquito pupae was calculated after all pupae eclosed or died. The number of adult mosquitoes relative to the total number of pupae is shown at the top of each column. The percentages are represented as the eclosion ratio of pupae.

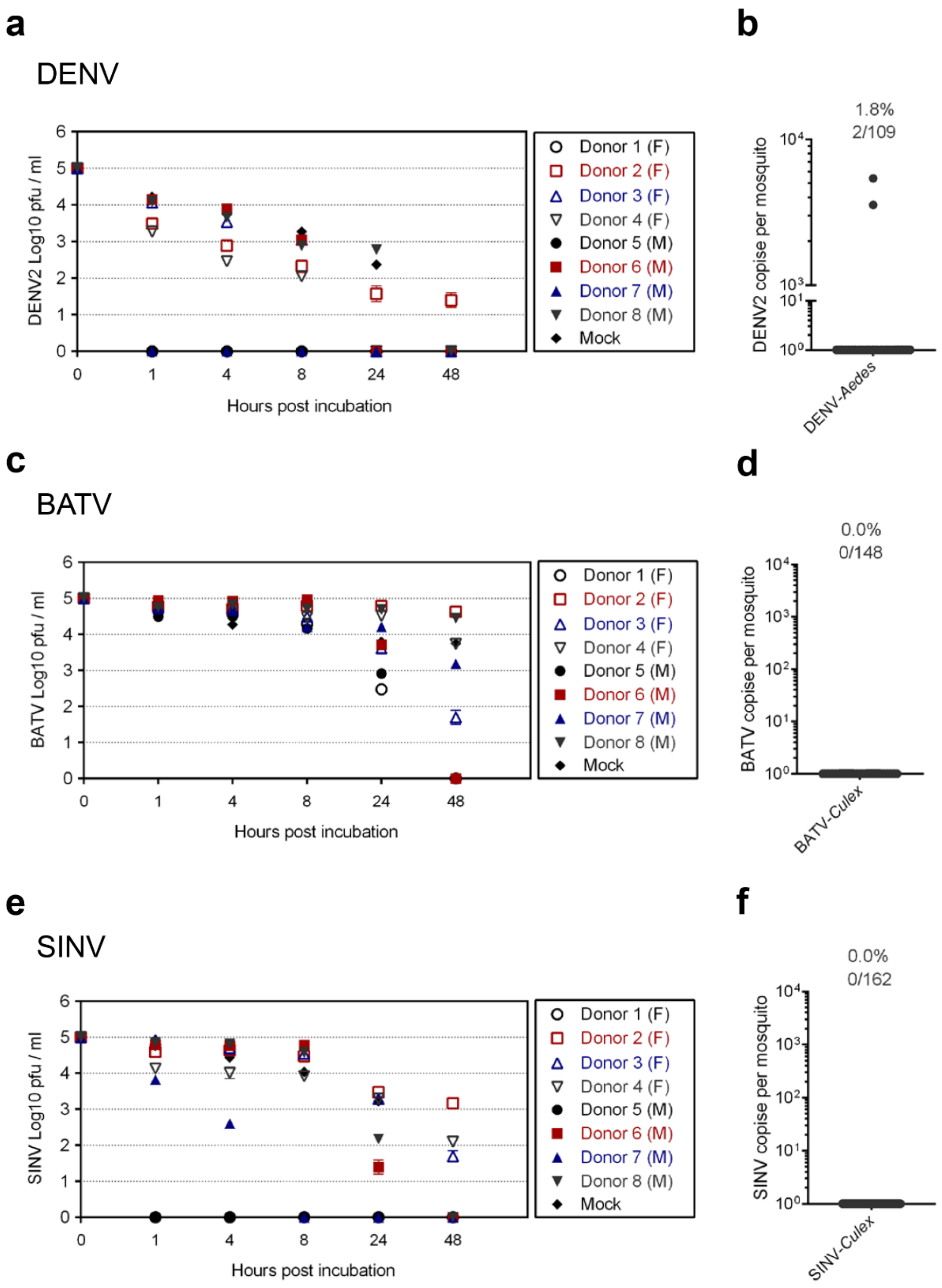

Supplementary Fig. 3 Arboviral infection in mosquitoes breeding from viral urine.

a, **c**, **e** Arboviral survivability in human urine. DENV2 (**a**), BATV (**c**) and SINV (**e**) were incubated with fresh human urine or PBS as control. The mixtures were then maintained at 37°C for a time course and subsequently subjected to a viral titration assay. The initial viral titer was 1×10^5 pfu/ml. The data represent 4 biologically independent samples from 2 parallel experiments. Data are presented as the mean ± S.E.M.

b, **d**, **f** Mosquito infection with various arboviruses. The pupae of the *A. aegypti* Rockefeller strain were reared in human urine (Donor 3) with DENV2 (**b**), while the pupae of *C. quinquefasciatus* Hainan strain were reared in the urine with BATV (**d**) or SINV (**f**). The final titer of each virus in the urine was 1×10^3 pfu/ml. The emerging adults were reared for 8 days for virus detection by RT-qPCR. The number of infected mosquitoes relative to the total number of mosquitoes is shown at the top of each column. One dot represents a mosquito. The percentages are represented as the ratios of mosquito infection.

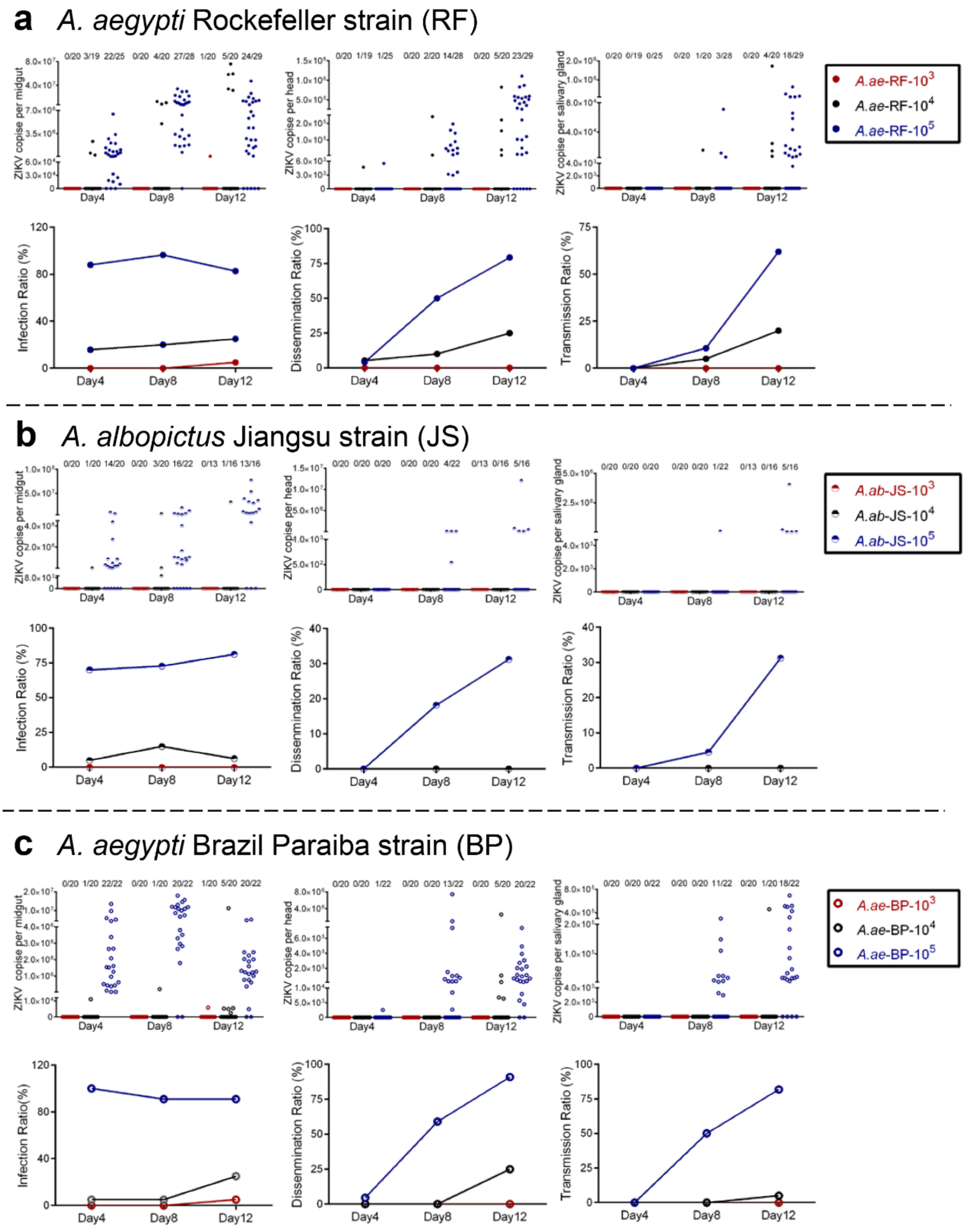

Supplementary Fig. 4 Assessing the vector competence of different *Aedes* strains after feeding on a serial titration of ZIKV. The mosquitoes, including the *A. aegypti* Rockefeller strain (**a**), the *A. albopictus* Jiangsu strain (**b**) and the *A. aegypti* Brazil Paraiba strain (**c**) were fed by human blood (50% v/v) and a supernatant from ZIKV infected Vero cells (50% /v). The ZIKV load was determined in the midgut, the head and the salivary glands at a time course by Taqman qPCR, respectively. The infection, dissemination, and transmission rates of

these mosquitoes were subsequently calculated accordingly. The upper panels: The number of infected mosquitoes relative to the total number of mosquitoes is shown at the top of each column. One dot represents a mosquito. The lower panels: The data represented the percentage of mosquito infection.

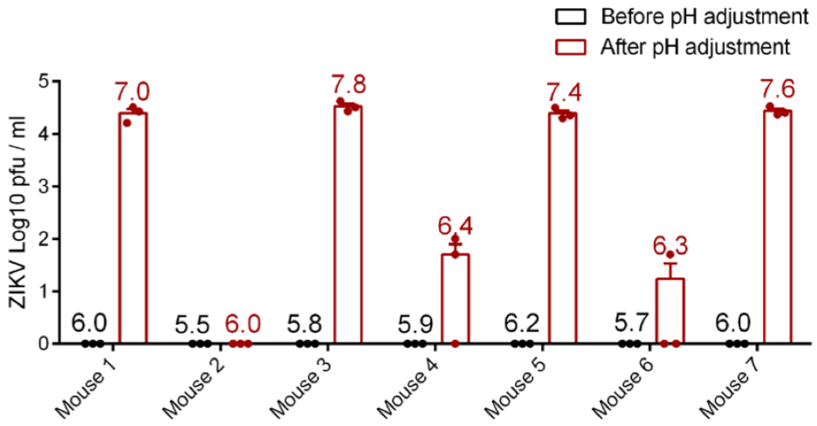

Supplementary Fig. 5 Adjustment of the pH value regulated ZIKV survivability in mouse urine.

The pH-manipulated urine samples were incubated with ZIKV supernatant from infected Vero cells (the initial titer 1×10^5 pfu /ml) for 4 hours at 37°C. The pH value is showed at the top of each column. Subsequently, the ZIKV titer was measured by a plaque assay. *n*=3 detection replicates. Data are presented as the mean ± S.E.M.

Supplementary Table 1. Literature for the *A. aegypti* and *A. albopictus* mosquitoes breeding in polluted water

Location	Study summary	Supplementary Reference
Brazil	*A. aegypti* can adapt to new sites and lay eggs in polluted water, such as the raw sewage.	1
Brazil	The proportion of cesspits found with *A. aegypti* immature forms in the two surveyed localities were as follows: Espigão do Oeste, 50.0% and Jaru, 27.3%. Moreover, the average number of insects did not differ significantly in the sampled cesspits and common breeding sites.	2
West Indies	Significant numbers of *A. aegypti* bred in septic tanks and underground drains. The primary producers of female mosquitoes were drums (165 per day), buckets (99 per day), tanks (63 per day) and septic tanks (too numerous to count).	3
Puerto Rico	The number of adult *A. aegypti* emerging per day from septic tanks in each community was 3 to 9 times larger than those produced in surface containers.	4
Puerto Rico	The *A. aegypti* larvae were present in sewage water and septic tanks (10.3 larvae per septic tank per day). The *A. aegypti* adults were recovered from 49% of the sampled tanks (8.7 adults per septic tank per day).	5
Puerto Rico	Underground aquatic habitats (septic tanks) produced large numbers of *A. aegypti* (>18,000 *A. aegypti* adults per day).	6
Mexico	*A. aegypti* and *C. Quinquefasciatus* were found breeding with notable frequency in all the different types of storm sewers (26.6% and 23.9%, respectively).	7
Malaysia	Seventy-two (55.4 percent) of the 130 septic tanks (contact filter-bed with pump sump and pump motor type) inspected had *A. albopictus* breeding.	8
Malaysia	The adaptation to poor and polluted water quality in dengue vectors mosquito larval habitat was related to pollutant from domestic and industrial exposure. Most of *A. albopictus* mosquitos preferred to breed in polluted water.	9
India	For both *A. aegypti* and *A. albopictus*, sewage drains were equally congenial habitat as were plastic, porcelain and earthen habitats.	10
Indonesia	*A.aegypti* mosquitoes bred well in polluted water in direct contact with the ground.	11
Sri Lanka	Both *A. aegypti* and *A. albopictus* preferred to breed in waste water with low dissolved oxygen levels and had a significantly high oviposition rate.	12
Nigeria	*C. quinquefasciatus*, *C. cinereus* and *A. aegypti* have been commonly found breeding in ammonia and nitrate-rich waters of latrines and septic tanks. These mosquitoes are able to breed in highly polluted water of septic tanks during the harsh dry months when most surface water bodies are dry.	13
Pakistan	Among 133 samples collected in wastewater irrigation system, 17.3% of the samples were positive for *Anopheles* larvae, 12.0% for *Culex* and 15.0% for *Aedes*.	14

Supplementary Table 2. Sewage samples information

Sample	Sample-#1	Sample-#2	Sample-#3	Sample-#4	Sample-#5	Sample-#6	Sample-#7	Sample-#8	Sample-#9	Sample-#10
**pH**	8.35	7.54	7.45	7.88	7.62	7.51	7.9	7.98	7.73	7.56
**COD (mg/L)**	80	172	232	349	462	780	1060	1285	1700	2300
**NH_3-N (mg/L)**	25	34	43	110	85	140	330	235	340	430

Supplementary Table 3. Sewage information of America

Location	Source	pH	COD (mg/L)	NH_3-N (mg/L)	Supplementary Reference
Curitiba-south-Brazil	Raw sewage	7.09 ±0.12	348.4±119.8	38 ±8.5	1
Sao Paulo- Brazil	Sewage	ND	274-588	ND	15.
Yamoussoukro-Coate d'Ivoire	Septic tank	7.0 ± 0.1	118.0±30.2	13.5 ±8.5	16
Sao Paulo- Brazil	Raw wastewater	ND	823	40.4	17
Brazil	Domestic sewage	6.7-8	450-800	20-35	18
Brazil	Domestic sewage	6.95 ±0.34	536±123.3	59.2 ±19.5	19
Port-au-Prince Haiti	Flush toilet	7.20 ± 0.06	1090 ± 90	164 ± 28	20.
Brazil	Raw sewage	7.13-7.17	213-1323	16-154	21.
Mexico	septic tank	7.9-8.5	740 ± 236	ND	22.

Supplementary Table 4. Human urine information

No.	Gender	Age	pH Value	Specific Gravity	Clarity	Color
1	Female	28	5.38	1.017	Clean	Yellow
2	Female	24	5.4	1.013	Clean	Yellow
3	Female	49	5.47	1.012	Clean	Light yellow
4	Female	11	5.76	1.009	Clean	Light yellow
5	Female	63	5.87	1.012	Clean	Light yellow
6	Female	24	5.94	1.006	Clean	Light yellow
7	Female	51	6.04	1.005	Clean	Light yellow
8	Female	64	6.06	1	Clean	Colorless
9	Female	47	6.2	1.005	Clean	Light yellow
10	Female	64	6.21	1.004	Clean	Light yellow
11	Female	77	6.38	1.007	Clean	Light yellow
12	Female	31	6.39	1	Clean	Colorless
13	Female	24	6.6	1.017	Clean	Yellow
14	Female	30	6.61	1.009	Clean	Light yellow
15	Female	11	6.66	1.004	Clean	Light yellow
16	Female	79	6.67	1.003	Clean	Light yellow
17	Female	39	6.74	1	Clean	Colorless
18	Female	23	6.81	1	Clean	Colorless
19	Female	60	6.84	1.011	Clean	Light yellow
20	Female	18	6.89	1.012	Clean	Light yellow
21	Female	25	6.9	1	Clean	Colorless
22	Female	44	6.92	1.01	Clean	Light yellow
23	Female	22	7.03	1.001	Clean	Colorless
24	Female	39	7.18	1	Clean	Colorless
25	Female	63	7.18	1.003	Clean	Colorless
26	Female	26	7.19	1.005	Clean	Light yellow
27	Female	16	7.39	1	Clean	Colorless
28	Female	73	7.48	1	Clean	Colorless
29	Male	36	5.29	1.02	Clean	Yellow
30	Male	27	5.32	1.021	Clean	Yellow
31	Male	44	5.35	1.004	Clean	Light yellow
32	Male	65	5.4	1.019	Clean	Yellow
33	Male	72	5.41	1.007	Clean	Light yellow
34	Male	56	5.44	1.016	Clean	Light yellow
35	Male	26	5.49	1.025	Clean	Yellow
36	Male	29	5.61	1.012	Clean	Light yellow
37	Male	9	5.63	1.02	Clean	Light yellow
38	Male	26	5.69	1.025	Clean	Yellow
39	Male	29	5.71	1.029	Clean	Yellow
40	Male	26	5.72	1.019	Clean	Light yellow
41	Male	25	5.78	1.023	Clean	Yellow
42	Male	42	5.8	1.013	Clean	Light yellow
43	Male	24	5.84	1.015	Clean	Yellow
44	Male	82	5.84	1.011	Clean	Yellow
45	Male	82	5.84	1.02	Clean	Yellow
46	Male	28	5.87	1.026	Clean	Yellow
47	Male	31	5.92	1.017	Clean	Yellow
48	Male	34	6.05	1.015	Clean	Light yellow
49	Male	30	6.1	1.016	Clean	Light yellow
50	Male	32	6.1	1.019	Clean	Light yellow
51	Male	32	6.1	1.02	Clean	Yellow
52	Male	52	6.12	1	Clean	Light yellow
53	Male	30	6.23	1.018	Clean	Light yellow
54	Male	34	6.25	1.017	Clean	Light yellow
55	Male	20	6.27	1.024	Clean	Light yellow
56	Male	26	6.27	1.011	Clean	Light yellow
57	Male	55	6.36	1.007	Clean	Light yellow
58	Male	26	6.37	1.004	Clean	Colorless
59	Male	61	6.41	1.023	Clean	Yellow
60	Male	44	6.47	1	Clean	Light yellow
61	Male	68	6.49	1	Clean	Colorless
62	Male	25	6.51	1.013	Clean	Light yellow
63	Male	34	6.55	1.021	Clean	Yellow
64	Male	54	6.56	1.004	Clean	Colorless
65	Male	21	6.6	1.012	Clean	Light yellow
66	Male	24	6.6	1.002	Clean	Light yellow
67	Male	43	6.72	1	Clean	Colorless
68	Male	43	6.75	1.012	Clean	Light yellow
69	Male	49	6.75	1	Clean	Light yellow
70	Male	21	6.8	1.004	Clean	Light yellow
71	Male	27	6.82	1	Clean	Colorless
72	Male	34	6.82	1	Clean	Colorless
73	Male	19	6.86	1	Clean	Light yellow
74	Male	25	6.88	1	Clean	Colorless
75	Male	22	7.05	1.007	Clean	Light yellow
76	Male	26	7.23	1.014	Clean	Light yellow
77	Male	25	7.27	1.004	Clean	Light yellow
78	Male	11	7.28	1	Clean	Light yellow
79	Male	22	7.29	1	Clean	Colorless
80	Male	15	7.42	1.003	Clean	Colorless
81	Male	77	7.65	1	Clean	Colorless

Supplementary Table 5. Primers and probes for PCR

**The primers for virus sequencing PCR**	**Upper primer**	**Lower primer**	
ZIKV fragment gene	AGGACAGGCCTTGAC	TGGCCATTCACATGT	
**The primers for Taqman RT-qPCR**	**Upper primer**	**Lower primer**	**Probe (for Taqman qPCR)**
ZIKV Envelope gene	CCGCTGCCCAACACAAG	CCACTAACGTTCTTTTGCAGACAT	FAM-AGCCTACCTTGACAAGCARTCAGACACTCAA-TAMRA
DENV2 Envelope gene	CATTCCAAGTGAGAATCTCTTTGTCA	CAGATCTCTGATGAATAACCAACG	FAM-ATGCTGAAACGCGAGAGAAACCGC-TAMRA
**The primers for SYBR RT-qPCR**	**Upper primer**	**Lower primer**	
BATV S gene	CAGTTCCAGACGATGGTCTTACC	GGTCACTCACTTTCAGAATCTTCTCA	
SINV Envelope1 gene	CGGCTATGGCAGGTTCGTA	CGCGCTTCAAGGACTTTTTC	

Supplementary References

1. Chitolina, R. F., Anjos, F. A., Lima, T. S., Castro, E. A. & Costa-Ribeiro, M. Raw sewage as breeding site to *Aedes* (Stegomyia) *aegypti* (Diptera, culicidae). *Acta Trop.* **164,** 290-296 (2016).
2. Herman Soares Gil, L. *et al.* Rudimentary cesspits as breeding sites for *Aedes aegypti* in urban areas of Northern Brazil. *Communication* **6,** 73-80 (2015).
3. Chadee, D. D. & Martinez, R. *Aedes aegypti* (L.) in Latin American and Caribbean region: With growing evidence for vector adaptation to climate change? *Acta Trop.* **156,** 137-143 (2016).
4. Mackay, A. J., Amador, M., Diaz, A., Smith, J. & Barrera, R. Dynamics of *Aedes aegypti* and *Culex quinquefasciatus* in septic tanks. *J. Am. Mosq. Control Assoc.* **25,** 409-416 (2009).
5. Burke, R., Barrera, R., Lewis, M., Kluchinsky, T. & Claborn, D. Septic tanks as larval habitats for the mosquitoes *Aedes aegypti* and *Culex quinquefasciatus* in Playa-Playita, Puerto Rico. *Med. Vet. Entomol.* **24,** 117-123 (2010).
6. Barrera, R. *et al.* Unusual productivity of *Aedes aegypti* in septic tanks and its implications for dengue control. *Med. Vet. Entomol.* **22,** 62-69 (2008).
7. Manrique-Saide, P. *et al.* Storm sewers as larval habitats for *Aedes aegypti* and *Culex spp*. in a neighborhood of Merida, Mexico. *J. Am. Mosq. Control Assoc.* **28,** 255-257 (2012).
8. Lam, W. K. & Dharmaraj, D. A survey on mosquitoes breeding in septic tanks in several residential areas around Ipoh municipality. *Med. J. Malaysia* **37,** 114-123 (1982).
9. Che Dom, N., Madzlan, F., Hasnan, A. & Misran, N. Water quality characteristics of dengue vectors breeding containers. *Int. J. Mosq. Res.* **25,** 25-29 (2016).
10. Banerjee, S. *et al.* Pupal productivity & nutrient reserves of *Aedes* mosquitoes breeding in sewage drains & other habitats of Kolkata, India: Implications for habitat expansion & vector management. *Indian J. Med. Res.* **142 Suppl,** S87-S94 (2015).
11. Utomo, S. W. & Triwibowo, G. A. *Aedes aegypti* Mosquito Breeding in Various Water Media. *Asian Journal of Applied Sciences* **2,** 481-487 (2016).
12. Gunathilaka, N., Ranathunge, T., Udayanga, L., Wijegunawardena, A. & Abeyewickreme, W. Oviposition preferences of dengue vectors; *Aedes aegypti* and *Aedes albopictus* in Sri Lanka

under laboratory settings. *Bull. Entomol. Res.* **108,** 442-450 (2018).

13. Nwoke, B. E., Nduka, F. O., Okereke, O. M. & Ehighibe, O. C. Sustainable urban development and human health: septic tank as a major breeding habitat of mosquito vectors of human diseases in south-eastern Nigeria. *Appl. Parasitol.* **34,** 1-10 (1993).

14. Mukhtar, M. *et al.* Role of wastewater irrigation in mosquito breeding in south Punjab, Pakistan. *Southeast Asian J. Trop. Med. Public Health* **34,** 72-80 (2003).

15. Marguti, A. L., Ferreira, F. S. & Piveli, R. P. Full-scale effects of addition of sludge from water treatment stations into processes of sewage treatment by conventional activated sludge. *J. Environ. Manage.* **215,** 283-293 (2018).

16. Kouame, P. K. *et al.* Ecohealth approach to urban waste management: exposure to environmental pollutants and health risks in Yamoussoukro, Cote d'Ivoire. *Int. J. Environ. Res. Public Health* **11,** 10292-10309 (2014).

17. Harada, H., Dong, N. T. & Matsui, S. A measure for provisional-and-urgent sanitary improvement in developing countries: septic-tank performance improvement. *Water Sci. Technol.* **58,** 1305-1311 (2008).

18. Von Sperling, M. & Chernicharo, C. A. L. Biological Wastewater Treatment in Warm Climate Regions. IWA Publishing, London, UK (2005).

19. de Sa, S. A., Marques, M., Severo, R. G. & Da, C. R. O. Engineered ecosystem for on-site wastewater treatment in tropical areas. *Water Sci. Technol.* **66,** 2131-2137 (2012).

20. Lansing, S., Maile-Moskowitz, A. & Eaton, A. Waste treatment and energy production from small-scale wastewater digesters. *Bioresour. Technol.* **245,** 801-809 (2017).

21. Beserra, E. B., Fernandes, C. R., Sousa, J. T., Freitas, E. M. & Santos, K. D. The effect of water quality in the life cycle and in the attraction for the egg oviposition of *Aedes aegypti* (L.) (Diptera: Culicidae). *Neotrop. Entomol.* **39**, 1016-1023 (2010).

22. Santiago-Diaz, A. L., Garcia-Albortante, J. & Salazar-Pelaez, M. L. UASB-septic tank as an alternative for decentralized wastewater treatment in Mexico. *Environ. Technol.* 1-13 (2018).
